# Prediction model of rehospitalization and mortality in heart failure patients with preserved and mildly reduced ejection fraction: the AD2NNER risk score

**DOI:** 10.3389/fcvm.2025.1605102

**Published:** 2025-07-25

**Authors:** Flavia-Mihaela Stoiculescu, Diana-Ruxandra Hădăreanu, Călin-Dinu Hădăreanu, Ionuț Donoiu, Octavian Istrătoaie, Victor-Cornel Raicea, Cristina Florescu

**Affiliations:** ^1^ Doctoral School, University of Medicine and Pharmacy of Craiova, Craiova, Romania; ^2^ Department of Cardiology, Clinical Emergency County Hospital of Craiova, Craiova, Romania; ^3^ Department of Cardiology, University of Medicine and Pharmacy of Craiova, Craiova, Romania; ^4^ Department of Cardiovascular Surgery, Clinical Emergency County Hospital of Craiova, Craiova, Romania; ^5^ Department of Cardiology, Filantropia Clinical Hospital of Craiova, Craiova, Romania

**Keywords:** heart failure, heart failure with mildly reduced ejection fraction, heart failure with preserved ejection fraction, rehospitalization, mortality, risk score

## Abstract

**Aims:**

This study aimed to identify predictors of heart failure (HF) rehospitalization and explore their association with mortality in patients with preserved (HFpEF), and mildly reduced (HFmrEF) ejection fraction, leading to the development of a multivariable risk prediction score.

**Methods:**

We enrolled 1,022 HFpEF and HFmrEF inpatients discharged between January 2019 and May 2023. Demographic, clinical, biological, and imaging data were collected for analysis.

**Results:**

After a mean follow-up of 3.5 ± 1.4 years, 308 (30.1%) patients experienced HF rehospitalization. Univariable analysis revealed several parameters associated with HF rehospitalization, including age (*p* < 0.001), male sex (*p* = 0.015), type 2 diabetes mellitus (T2DM, *p* = 0.016), arterial hypertension (*p* = 0.018), smoking (*p* = 0.029), NYHA class at discharge (*p* = 0.006), atrial fibrillation (*p* = 0.003), ischemic or congenital etiology (*p* = 0.011), serum sodium (*p* = 0.002), and several echocardiographic measures. Multivariate Cox regression revealed six independent predictors: age (HR = 0.98, *p* < 0.001), T2DM (HR = 1.31, *p* = 0.026), NYHA class (HR = 1.39, *p* = 0.010), ischemic or congenital etiology (HR = 1.33, *p* = 0.037), atrial fibrillation (HR = 0.65, *p* = 0.001), and serum sodium level (HR = 0.97, *p* = 0.005). These formed the AD2NNER (age, T2DM, serum natrium, NYHA class, etiology, rhythm) score, ranging from 0 to 9 points. Kaplan–Meier analysis confirmed reduced event-free survival in patients with scores ≥4 (log-rank *p* = 0.005). Comparative Kaplan–Meier curves using an unweighted risk count (0–6) showed less distinct stratification. Subgroup analysis revealed robust score performance in HFpEF, but not HFmrEF alone. Higher AD2NNER scores were also associated with all-cause mortality.

**Conclusion:**

The AD2NNER risk score is a simple, six-variable model that effectively predicts rehospitalization, and is also associated with mortality in patients with HFpEF and HFmrEF.

## 1 Introduction

Heart failure (HF) remains the leading cause of hospitalization among individuals aged 65 years and older and is characterized by high morbidity and mortality, along with a significant reduction in both quality of life and life expectancy, with a five-year survival rate of approximately 50% (1). Despite advancements in pharmacological and invasive therapies, the prevalence of HF remains high, and frequent rehospitalizations continue to impose a heavy social and economic burden. Recent epidemiologic trends underscore the urgent need to develop targeted strategies to reduce these recurrent hospital admissions (1).

Within the population diagnosed with HF, subgroups of patients present significant heterogeneity in key clinical aspects (2). This variability in presentation and pathophysiology plays a crucial role in influencing both prognosis and treatment strategies. Notably, patients with HF with mildly reduced ejection fraction (HFmrEF) have a clinical prognosis similar to that of patients with HF with preserved ejection fraction (HFpEF) (3), despite having etiologies and population characteristics comparable to those of patients diagnosed with HF with reduced EF (4).

Several risk prediction models have been developed to estimate outcomes in patients with HF, including CHARM (5), MAGGIC(6), I-PRESERVE(7), 3A3B (8), and WATCH-DM (9). However, many of these models are limited in scope, as they focus on the general HF population without adequately reflecting the prognosis of hospitalized patients with HFpEF and HFmrEF, rely on data from older trials or registries, or lack some important variables. Additionally, the majority of risk prediction models designed for patients with HFpEF primarily address mortality (5–8), despite evidence that each rehospitalization further worsens long-term prognosis (9).

Accurately predicting patients’ prognosis in HF patients is essential for tailoring personalized care strategies and improving outcomes (6). Accordingly, the objective of this study was to indentify clinical predictors of HF rehospitalization in patients with HFpEF and HFmrEF, and to evaluate their potential prognostic value for mortality, leading to the development of a practical multivariable risk score. This tool is designed to enhance personalized care by accurately identifying patients at high risk of rehospitalization and death, ultimately guiding targeted interventions and improving patient outcomes.

## 2 Methods

### 2.1 Study design

**Figure 1:**
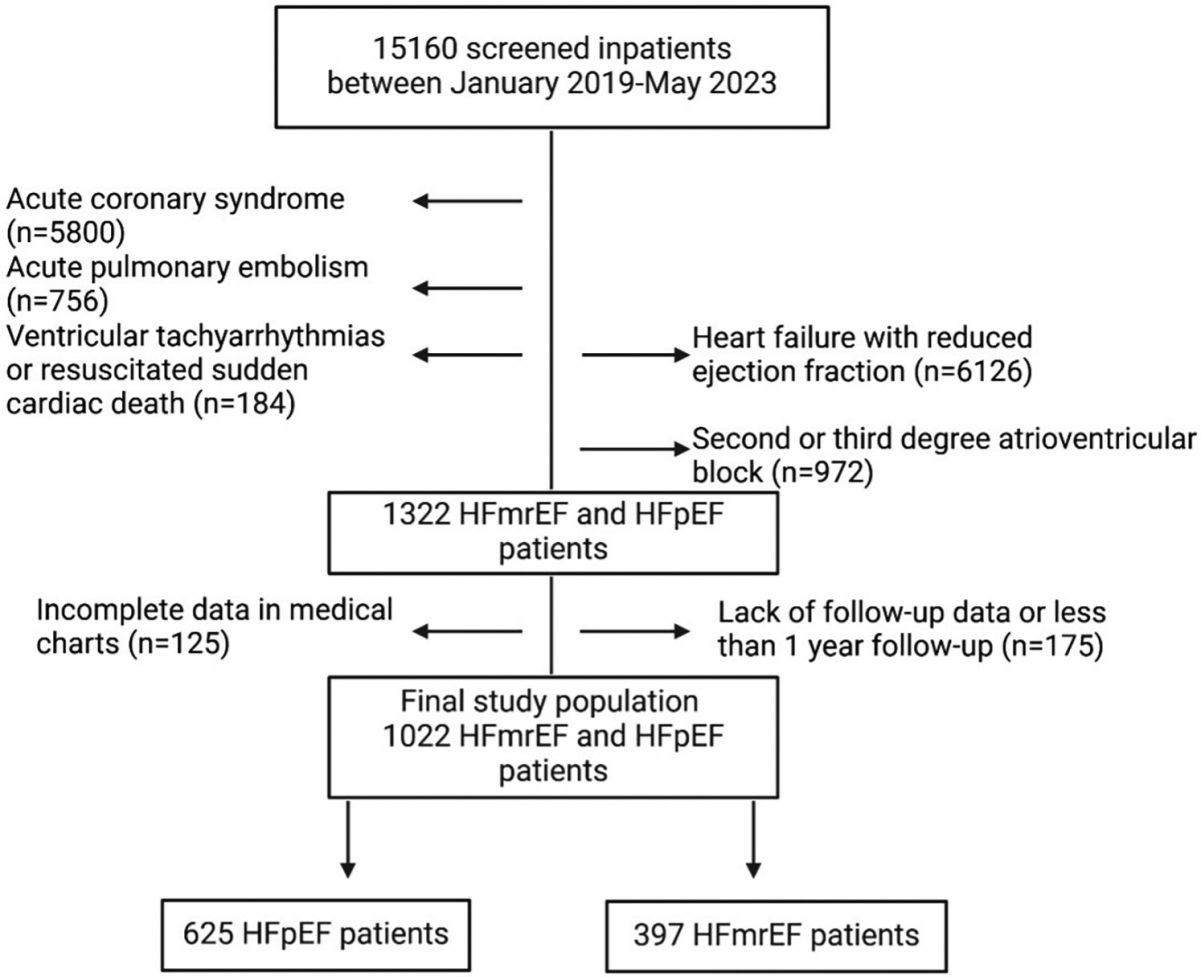
Flowchart describing the patient selection process.

To fulfill our aims, 15,160 consecutive patients discharged between 1 January 2019 and 31 May 2023 from Clinical Emergency County Hospital of Craiova, Romania were retrospectively screened. Patients with a first diagnosis of either HFpEF or HFmrEF at hospital discharge (4) were included in the analysis. The exclusion criteria were one of the following diagnoses during the index hospitalization: (i) acute coronary syndrome (*n* = 5800), (ii) acute pulmonary embolism (*n* = 756), (iii) ventricular tachyarrhythmia or resuscitated sudden cardiac death (*n* = 184), (iv) heart failure with a reduced ejection fraction (*n* = 1,626), (v) second- or third-degree atrioventricular block (*n* = 972), (vi) incomplete data in medical charts (*n* = 125), and (vii) lack of follow-up data or less than 1 year of follow-up (*n* = 175). The flowchart describing the patient selection process is shown in Figure 1. The study was approved by the Ethics Committee of our institution (Approval No. 86/19.02.2024) and was conducted in accordance with the Declaration of Helsinki. Owing to the retrospective nature of the study, written informed consent from patients was waived, and the data were anonymized prior to inclusion in the analysis.

### 2.2 Data collection

Demographic and clinical data, including age; sex; cardiac rhythm; heart rate; blood pressure; New York Heart Associations (NYHA) class at discharge; the presence of cardiovascular risk factors; and available biochemical, echocardiography, medication and past medical history, were recorded for all the subjects included in the study.

The biochemical data recorded included hemoglobin, alanine transaminase, aspartate transaminase, serum creatinine, sodium and potassium values, and N-terminal prohormone of brain natriuretic peptide (NT-proBNP) levels. The estimated glomerular filtration rate (eGFR) was calculated via the Chronic Kidney Disease Epidemiology Collaboration (CKD-EPI) formula.

The echocardiographic data available and included in the analysis were left and right ventricular and atrial linear dimensions, tricuspid regurgitation jet maximum velocity and estimated systolic pulmonary artery pressure (sPAP), left ventricular (LV) ejection fraction (EF), tricuspid annulus plane systolic excursion (TAPSE), the ratio between the E and A pulsed-Doppler waves at the level of the mitral leaflet tips, and the presence or absence of valvular heart disease. All echocardiographic measurements were performed according to current guidelines of the European Association of Cardiovascular Imaging. LVEF was determined using the Simpson's biplane method and reconfirmed at the time of hospital discharge (10). Left and right chamber dimensions were assessed in standard parasternal and apical views (10), and TAPSE by M-mode was calculated from the apical 4-chamber view (10). SPAP was calculated from the maximal velocity of the tricuspid regurgitant jet and right atrial pressure estimate from inferior vena cava size and inspiratory collapsibility index (11). Moreover, the LV relative wall thickness (RWT) was calculated via the formula LV posterior wall width*2/LV end-diastolic diameter, and the LV mass (LVM) was calculated via the Devereaux formula, and normal LV geometry, concentric remodeling or hypertrophy or eccentric hypertrophy were defined on the basis of sex-specific cutoff values of the LV mass and RWT (≤ or >0.42).

The conventional cardiovascular risk factors were as follows: (1) current or past history of smoking, (2) dyslipidemia (LDL-cholesterol >130 mg/dl or taking lipid lowering therapies), (3) arterial hypertension (repeated blood pressure values of ≥140/90 mmHg or taking antihypertensive drugs), (4) type 2 diabetes mellitus (T2DM), and (5) chronic kidney disease.

Most patients with suspected ischemic heart disease underwent diagnostic coronary angiography. CT coronary angiography was rarely used and not systematically recorded. An ischemic etiology of HF was considered in patients with evidence of either significant coronary artery stenosis at angiography (>70% single vessel disease or >50% left main stenosis) or prior coronary artery revascularization. or a history of previous myocardial infarction. Patients with prior coronary angioplasty but no infarction were also classified as ischemic. Coronary angiography was performed based on clinical judgment, and in patients without clear ischemic documentation but low clinical suspicion, invasive assessment was often deferred. As a result, some ischemic cases may have remained undiagnosed. Valvular heart disease was defined as any hemodynamically significant stenosis or regurgitation (at least moderate in severity). Congenital heart disease included any congenital cardiovascular malformation, such as atrial septal defect, bicuspid aortic valve, ventricular septal defect, Ebstein disease, atrioventricular septal defect, incomplete atrioventricular canal, and repaired tetralogy of Fallot.

To ensure completeness of data, patients with missing or incomplete baseline clinical, laboratory, or echocardiographic variables were excluded from the final analysis (*n* = 125, Figure 1).

### 2.3 Follow-up and study endpoints

Rehospitalization for HF decompensation was the chosen primary endpoint. The secondary end-point was all-cause death. The information about survival and rehospitalization was gathered from the medical records of either outpatient visits or hospital admissions, as well as phone conversations with patients or family members. Mortality status was independently confirmed via the national identification number of each patient, and HF rehospitalization was defined as hospital admission for a primary diagnosis of HF. For prognostic analysis, the date of last contact was used for patients without events, while the date of the first HF rehospitalization was used for those with multiple events.

### 2.4 Statistical analysis

The normality of the distribution of continuous variables was assessed via the Shapiro–Wilk test. Data are reported as the means ± standard deviations (SDs) for continuous variables and as numbers and percentages for categorical variables. Continuous variables were compared via Student's *t* test, and categorical variables were compared via the chi-square test. Univariable Cox proportional hazard regression analysis was used to determine the associations between the clinical, biochemical and echocardiographic data and the chosen endpoint. Several multivariable Cox proportional hazard models, including the parameters that were statistically significant in the univariable analysis (*p* < 0.05), were computed to determine the parameters that remained independently associated with the outcome. Before the multivariable analysis, the variance inflation factor was determined to exclude multicollinearity, and values between 1 and 10 were considered the absence of collinearity. The variables with statistical significance in the multivariable Cox regression analysis were included in a risk score, and the number of points given to each of them was calculated by dividing the *χ*² of each of them by that of the parameter with the lowest *χ*² value. Time-dependent receiver operating characteristic (ROC) curve analysis was performed to assess the associations of both continuous variables and the computed score with the endpoint, and the optimal cutoffs to predict the outcome were chosen on the basis of Youden's J index. Kaplan–Meier survival analysis was then used to evaluate the event-free survival rates, and the differences between the event-free survival curves of the population groups dichotomized on the basis of the chosen cutoff value of the risk score were analyzed via the log-rank test. Additionally, a Kaplan–Meier survival analysis was performed with patients stratified into ten groups according to their individual AD2NNER scores (0–9) to evaluate the stepwise discriminatory capacity of the score. To further explore additive risk, a separate Kaplan–Meier analysis was performed by stratifying patients based on the unweighted number of individual risk factors present (ranging from 0 to 6). Log-rank testing was used to compare cumulative event-free survival across all groups. Finally, subgroup analyses were conducted for patients with HFmrEF and HFpEF to determine whether the AD2NNER score and associated predictors performed differently based on LVEF category. Kaplan–Meier survival curves were generated separately within each group using the full AD2NNER score (0–9), the dichotomized score (0–3 vs. ≥4), and the unweighted risk factor count (0–6).

Binary logistic regression analysis was conducted to assess the association between the six predefined AD2NNER risk factors and all-cause mortality, given the lack of time-to-death data. Univariable models were first performed for each predictor individually. A multivariable logistic regression model including all six variables (age ≥75 years, NYHA class III–IV, AF, T2DM, hyponatremia, and ischemic or congenital etiology) was subsequently used to determine their independent association with mortality. Additional models assessed the predictive capacity of the total AD2NNER score (analyzed both as a continuous variable and dichotomized at ≥4), as well as the unweighted count of risk factors present (range 0–6). The statistical analysis was performed via SPSS version 23 for Mac (SPSS Inc., IBM Corp., Chicago, IL), and a two-sided *p* value <0.05 was considered significant.

## 3 Results

### 3.1 Baseline characteristics of the study population

Among the 15,160 patients screened, 1,022 patients with HFpEF (*n* = 625, 61%) and HFmrEF (*n* = 397, *n* = 38.9%) were included. Table 1 lists the characteristics of the study population. Of note, patients with atrial flutter were included in the ‘atrial fibrillation’ group due to shared clinical implications. Among the 512 patients categorized as having atrial fibrillation, 22 had atrial flutter. During a mean follow-up of 3.5 ± 1.4 years, 308 (30.1%) patients experienced at least one HF rehospitalization. Patients who met the endpoint more frequently had an ischemic etiology of HF or congenital heart disease (*p* < 0.001) than did those who did not meet the endpoint. Regarding HF etiology, the most prevalent etiologies were hypertensive heart diseases, valvular heart diseases, and pulmonary arterial hypertension (Table 1). Moreover, these patients had a greater incidence of arterial hypertension (*p* = 0.031), T2DM (*p* = 0.048), and atrial fibrillation (AF, *p* = 0.009) and were more symptomatic (higher NYHA class) at discharge (*p* = 0.006). Finally, the patients who experienced HF rehospitalization had lower values of serum sodium (*p* = 0.004), LV EF (*p* = 0.048) and TAPSE (*p* = 0.004) and greater LV (*p* = 0.025), right ventricular (*p* = 0.003), left atrial (*p* < 0.001), and right atrial (*p* < 0.001) maximum diameters. No statistically significant difference was found regarding the pattern of LV geometrical remodeling or severity of valvular regurgitation between the two groups.

**Table 1:** Characteristics of the study population.

Parameter	Entire study population (*n* = 1,022)	Patients with rehospitalizations for HF (*n* = 308)	Patients without rehospitalizations for HF (*n* = 714)	*p* value^a^
Clinical				
Age (years)	74 ± 11	72 ± 11	74 ± 11	0.006
Men, *n* (%)	518 (50.7%)	174 (56.5%)	344 (48.2%)	0.013
Heart rate (beats per minute)	73 ± 14	72 ± 12	73 ± 14	0.169
Systolic blood pressure (mmHg)	130 ± 20	130 ± 20	130 ± 20	0.876
Diastolic blood pressure (mmHg)	76 ± 11	75 ± 11	76 ± 11	0.582
Heart failure etiology				<0.001
Hypertensive heart disease, *n* (%)	385 (37.7%)	97 (31.5%)	288 (39.2%)	
Primary cardiomyopathies, *n* (%)	23 (2.3%)	7 (2.3%)	16 (5.1%)	
Ischemic heart disease, *n* (%)	181 (17.7%)	61 (19.8%)	120 (16.8%)	
Valvular heart disease, *n* (%)	262 (25.6%)	77 (25%)	185 (25.9%)	
Pulmonary hypertension/Cor pulmonare, *n* (%)	147 (14.4%)	49 (15.9%)	98 (13.7%)	
Congenital heart disease, *n* (%)	24 (2.3%)	17 (5.5%)	7 (0.98%)	
Conventional cardiovascular risk factors				
Type 2 Diabetes Mellitus *n* (%)	375 (36.7%)	127 (41.2%)	248 (34.7%)	0.048
Hypertension, *n* (%)	806 (78.9%)	230 (74.7%)	576 (80.67%)	0.031
Hypercholesterolemia, *n* (%)	663 (64.9%)	203 (65.9%)	460 (64.4%)	0.649
Smoking *n* (%)	141 (13.8%)	52 (16.9%)	90 (12.6%)	0.074
Chronic kidney disease, *n* (%)	347 (34%)	107 (34.7%)	240 (33.6%)	0.727
eGFR < 30 ml/min/1.73 m^2^, *n* (%)	52 (5.1%)	14 (4.6%)	38 (5.3%)	0.604
NYHA class				0.006
2, *n* (%)	444 (43.4%)	111 (36%)	333 (46.63%)	
3, *n* (%)	492 (48.1%)	170 (55.2%)	322 (45.09%)	
4, *n* (%)	86 (8.5%)	30 (9.7%)	56 (7.8%)	
Cardiac rhythm				0.009
Sinus, *n* (%)	510 (49.9%)	130 (42.20%)	380 (53.22%)	
Atrial fibrillation, *n* (%)	512 (50.1%)	178 (57.8%)	334 (48.8%)	
Medical treatment				
Angiotensin-converting enzyme inhibitors/Angiotensin receptor-neprilysin inhibitor, *n* (%)	666 (65.2%)	189 (61.3%)	477 (66.8%)	0.094
Beta-blockers, *n* (%)	857 (83.9%)	261 (84.74%)	596 (83.47%)	0.614
Sodium-glucose cotransporter 2 inhibitors, *n* (%)	76 (7.4%)	30 (9.74%)	46 (6.44%)	0.076
Mineralocorticoid receptor antagonists, *n* (%)	661 (64.5%)	206 (66.88%)	455 (63.72%)	0.189
Diuretics, *n* (%)	850 (83.2%)	266 (86.36%)	584 (81.79%)	0.159
Laboratory data				
NT-proBNP (pg/ml)	5,543 ± 7,524	5,565 ± 7,943	5,534 ± 7,379	0.971
LDL-cholesterol (mg/dl)	89 ± 37	84 ± 36	91 ± 37	0.003
Creatinine (mg/dl)	1.2 ± 0.7	1.21 ± 0.66	1.2 ± 0.72	0.801
Glucose (mg/dl)	123 ± 54	126 ± 61	122 ± 52	0.235
Hemoglobin (g/dl)	13.5 ± 1	13.4 ± 0,4	13.5 ± 1.4	0.234
Aspartate transaminase (U/L)	39 ± 162	50 ± 294	34 ± 27	0.163
Alanine transaminase (U/L)	33 ± 96	37.7 ± 168	30 ± 36	0.270
Serum potassium (mmol/L)	4.5 ± 0.7	4.5 ± 0.6	4.5 ± 0.7	0.335
Serum sodium (mmol/L)	138 ± 6	137 ± 4.6	138 ± 6	0.004
Echocardiographic data				
Interventricular septum (mm)	13 ± 2	13 ± 2	13 ± 2	0.947
Left ventricular diastolic diameter (mm)	50 ± 7	50 ± 7	49 ± 7	0.025
Left ventricular posterior wall width (mm)	12 ± 2	12 ± 2	12 ± 2	0.309
Left ventricular mass (g)	241 ± 78	248 ± 78	238 ± 76	0.081
Left ventricular relative wall thickness	0.49 ± 0.13	0.49 ± 0.13	0.49 ± 0.13	0.663
Left atrial diameter (mm)	48 ± 10	50 ± 9	47 ± 10	<0.001
Mitral E/A ratio	1.3 ± 1	3.1 ± 0.6	3 ± 0.7	0.052
Tricuspid regurgitation jet maximum velocity (m/s)	3 ± 0.7	52 ± 18	50 ± 17	0.459
Systolic pulmonary artery pressure (mmHg)	51 ± 17	52 ± 18	50 ± 17	0.242
Right atrial diameter (mm)	43 ± 9	45 ± 10	42 ± 8	<0.001
Tricuspid annulus plane systolic excursion (mm)	19 ± 5	18 ± 4	19 ± 5	0.004
Right ventricular basal diameter (mm)	37 ± 8	38 ± 8	37 ± 7	0.003
Left ventricular ejection fraction (%)	49 ± 6	49 ± 6	50 ± 6	0.048
Tricuspid regurgitation severity				0.683
Trace/mild	484 (47.4%)	138 (44.8%)	346 (48.4%)	
Moderate	283 (27.7%)	87 (28.2%)	196 (27.4%)	
Severe	254 (24.9%)	83 (26.9%)	171 (23.9%)	
Mitral regurgitation severity				0.578
No/mild	429 (42%)	120 (38.9%)	306 (42.8%)	
Moderate	366 (35.8%)	117 (37.9%)	249 (34.8%)	
Severe	82 (22.2%)	69 (22.4%)	158 (22.1%)	
Aortic regurgitation severity				0.272
No/mild	396 (38.7%)	119 (38.6%)	277 (38.7%)	
Moderate	126 (12.3%)	31 (10.0%)	95 (13.3%)	
Severe	31 (3%)	7 (2.2%)	13 (1.8%)	
LV geometry				0.052
Normal LV geometry	118 (11.5%)	36 (11.6%)	82 (11.4%)	
LV concentric remodeling	313 (30.6%)	83 (26.9%)	230 (32.2%)	
LV concentric hypertrophy	428 (41.9%)	137 (44.4%)	291 (40.7%)	
LV eccentric hypertrophy	162 (15.9%)	51 (16.5%)	111 (15.5%)	

The data are expressed as the means ± standard deviations.

^a^ *T* test for continuous variables or chi-square test for categorical variables. eGFR, estimated glomerular filtration rate; NYHA, New York Heart Association.

### 3.2 Association of data with the endpoint of HF rehospitalization

The results of the univariable Cox regression analysis for the parameters included in Table 1 are summarized in Table 2. The clinical variables predictive of the primary endpoint in the univariable analysis were age, male sex, T2DM status, hypertension status, smoking status, NYHA class at discharge, cardiac rhythm and etiology of HF (ischemic or congenital). Among the biochemical and echocardiographic parameters, aspartate transaminase, serum sodium, the maximum diameter of all four cardiac chambers and the TAPSE were associated with the outcome.

**Table 2:** Univariable Cox regression analysis for the primary endpoint.

Parameter	HR (95% CI)	*P* value
Age (years)	0.98 (0.97–0.99)	<0.001^a^
Heart rate (beats per minute)	0.99 (0.99–1.01)	0.256
Gender	0.75 (0.60–0.95)	0.015^a^
Systolic blood pressure	0.99 (0.99–1.01)	0.650
Diastolic blood pressure	0.99 (0.98–1.01)	0.646
Ischemic or congenital etiology	1.39 (1.08–1.80)	0.011
Cardiac rhythm (sinus vs. atrial fibrillation)	0.71 (0.56–0.89)	0.003^a^
Type 2 diabetes mellitus	1.32 (1.05–1.66)	0.016^a^
Hypertension	0.73 (0.56–0.95)	0.018^a^
Hypercholesterolemia	0.98 (0.78–1.25)	0.916
Smoking	1.21 (1.02–1.43)	0.029^a^
Chronic kidney disease	1.03 (0.82–1.31)	0.792
eGFR < 30 ml/min/1.73 m^2^	0.91 (0.53–1.55)	0.718
NYHA class 3–4 vs. 1–2	1.28 (1.07–1.52)	0.006^a^
NT-proBNP	1 (0.99–1.01)	0.866
Creatinine	1.03 (0.88–1.20)	0.738
eGFR	1.00 (0.96–1.01)	0.905
Hemoglobin	1.00 (0.99–1.01)	0.224
Aspartate transaminase	1.00 (1.00–1.01)	0.021^a^
Alanine transaminase	1.01 (1.00–1.01)	0.057
Serum sodium	0.97 (0.95–0.99)	0.002^a^
Serum potassium	1.05 (0.88–1.25)	0.601
Left ventricular mass	1.01 (1–1.01)	0.150
Left ventricular relative wall thickness	0.73 (0.31–1.72)	0.464
Left ventricular diastolic diameter	1.02 (1.00–1.04)	0.023^a^
Left atrial diameter	1.02 (1.01–1.03)	<0.001^a^
Systolic pulmonary artery pressure	1.00 (0.99–1.02)	0.290
Tricuspid regurgitation jet maximum velocity	1.15 (0.81–1.63)	0.442
Tricuspid regurgitation (moderate/severe vs. mild/trace)	1.20 (0.96–1.51)	0.109
Mitral regurgitation (moderate/severe vs. mild/no)	1.17 (0.93–1.47)	0.177
Eccentric hypertrophy vs. normal left ventricular geometry or concentric remodeling/hypertrophy	1.08 (0.80–1.46)	0.619
Right atrial diameter	1.02 (1.01–1.04)	<0.001^a^
Tricuspid annulus plane systolic excursion	0.94 (0.89–0.98)	0.007^a^
Right ventricular basal diameter	1.02 (1.01–1.04)	0.001^a^
Left ventricular ejection fraction	0.98 (0.97–1.00)	0.05
Mitral E/A ratio	1.14 (0.90–1.31)	0.074

The data are expressed as the means ± standard deviations.

^a^ *T* test for continuous variables or chi-square test for categorical variables. eGFR, estimated glomerular filtration rate; NYHA, New York Heart Association.

Furthermore, after excluding any collinearity between the tested parameter variables that were significant in the univariable analysis (*p* < 0.05), a multivariable Cox regression analysis was computed to test the independent associations between the clinical and biochemical variables and the primary endpoint (Table 3). In the multivariable analysis, the variables that remained independently associated with the primary endpoint were age (*p* < 0.001), T2DM status (*p* = 0.026), NYHA class (*p* = 0.010), etiology of HF (*p* = 0.037), cardiac rhythm (*p* = 0.001) and serum sodium levels (*p* = 0.005, Table 3).

**Table 3:** Multivariate cox regression analysis for the primary endpoint.

Parameter	*χ*²	HR (95% CI)	*P* value
Age	12.171	0.98 (0.97–0.99)	<0.001^a^
Gender		0.86 (0.68–1.10)	0.224
Type 2 diabetes mellitus	5.841	1.31 (1.03–1.65)	0.026^a^
Hypertension		0.93 (0.71–1.24)	0.632
Smoking		1.15 (0.956–1.38)	0.123
NYHA class	10.123	1.39 (1.08–1.78)	0.010^a^
Ischemic or congenital etiology	6.461	1.33 (1.02–1.74)	0.037^a^
Cardiac rhythm (sinus vs. atrial fibrillation)	9.129	0.65 (0.51–0.84)	0.001^a^
Serum sodium	7.774	0.97 (0.94–0.99)	0.005^a^
Aspartate transaminase		0.81 (1.00–1.01)	0.081

The data are expressed as the means ± standard deviations.

^a^ *T* test for continuous variables or chi-square test for categorical variables. eGFR, estimated glomerular filtration rate; NYHA, New York Heart Association.

### 3.3 Computation of the AD2NNER (age, T2DM, serum natrium, NYHA class, etiology of HF, rhythm) risk score for the primary endpoint of HF rehospitalization

**Figure 2:**
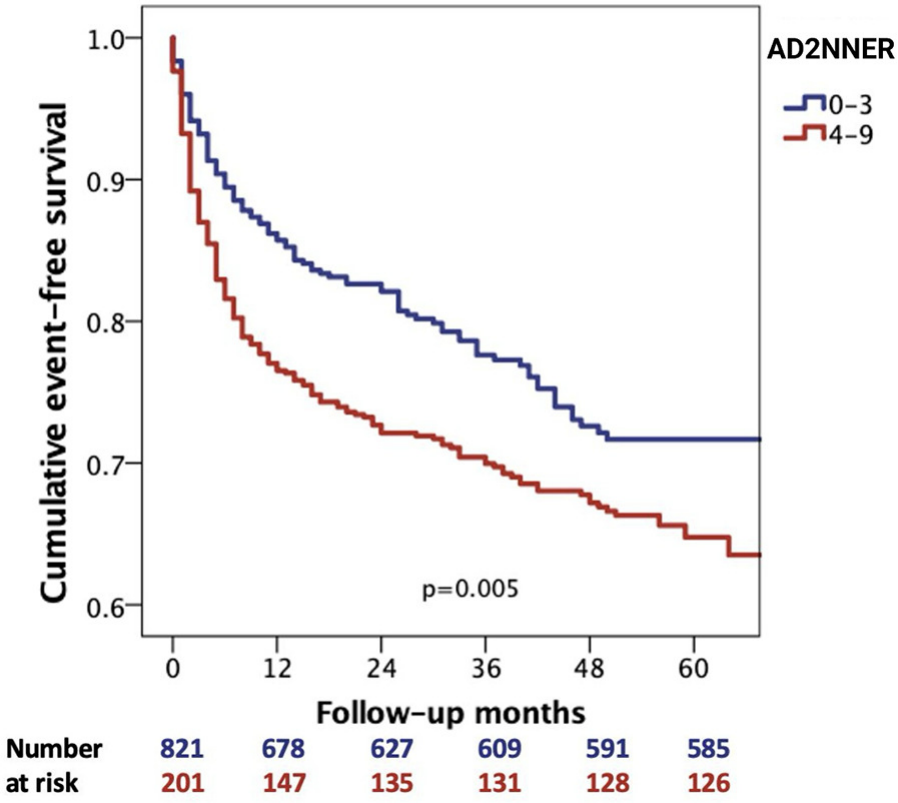
Kaplan–Meier analysis for cumulative event-free survival according to AD2NNER scores <4 and ≥4.

The variables that remained independently associated in the multivariable Cox regression analysis were combined in a rehospitalization risk score, with 2 points given for age ≥75 years, NYHA class 3 or 4, and AF, and 1 point for each of the T2DM, hyponatremia (serum sodium <135 mmol/L), and ischemic or congenital etiologies of HF. The scoring system ranged from a minimum of 0 points to a maximum of 9 points. Furthermore, according to the Youden's J index derived from the ROC curve, the best cutoff for predicting the primary outcome was 3.5 points. Subsequently, Kaplan–Meier curves were derived for the event-free survival evaluation according to score values of less or more than 3.5 (Figure 2). We found that HFpEF and HFmrEF patients with a score of 4 or more had a significantly lower probability of event-free survival (log-rank *p* = 0.005).

**Figure 3:**
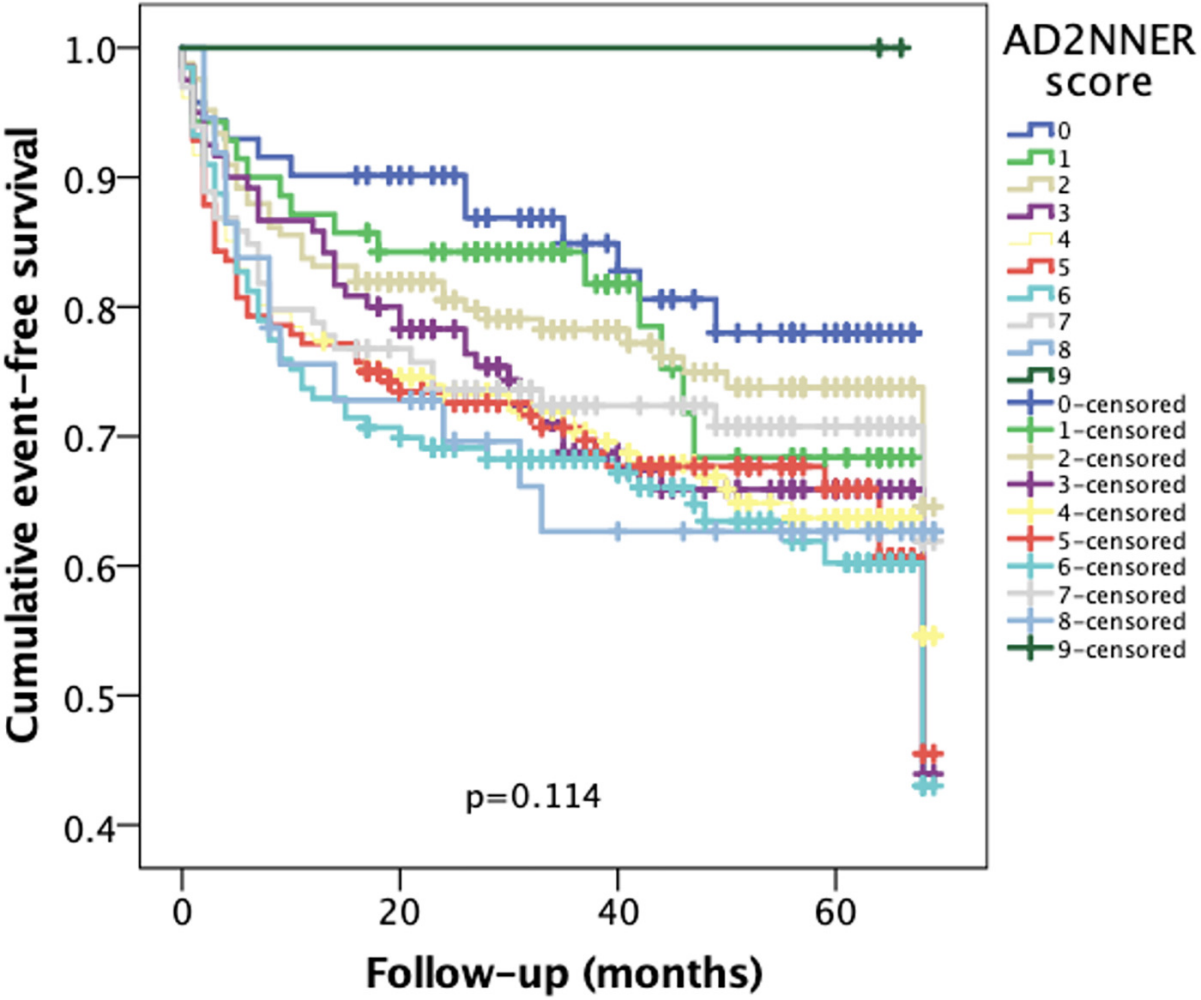
Kaplan–Meier curves showing cumulative event-free survival stratified by individual AD2NNER scores (0–9).

**Figure 4:**
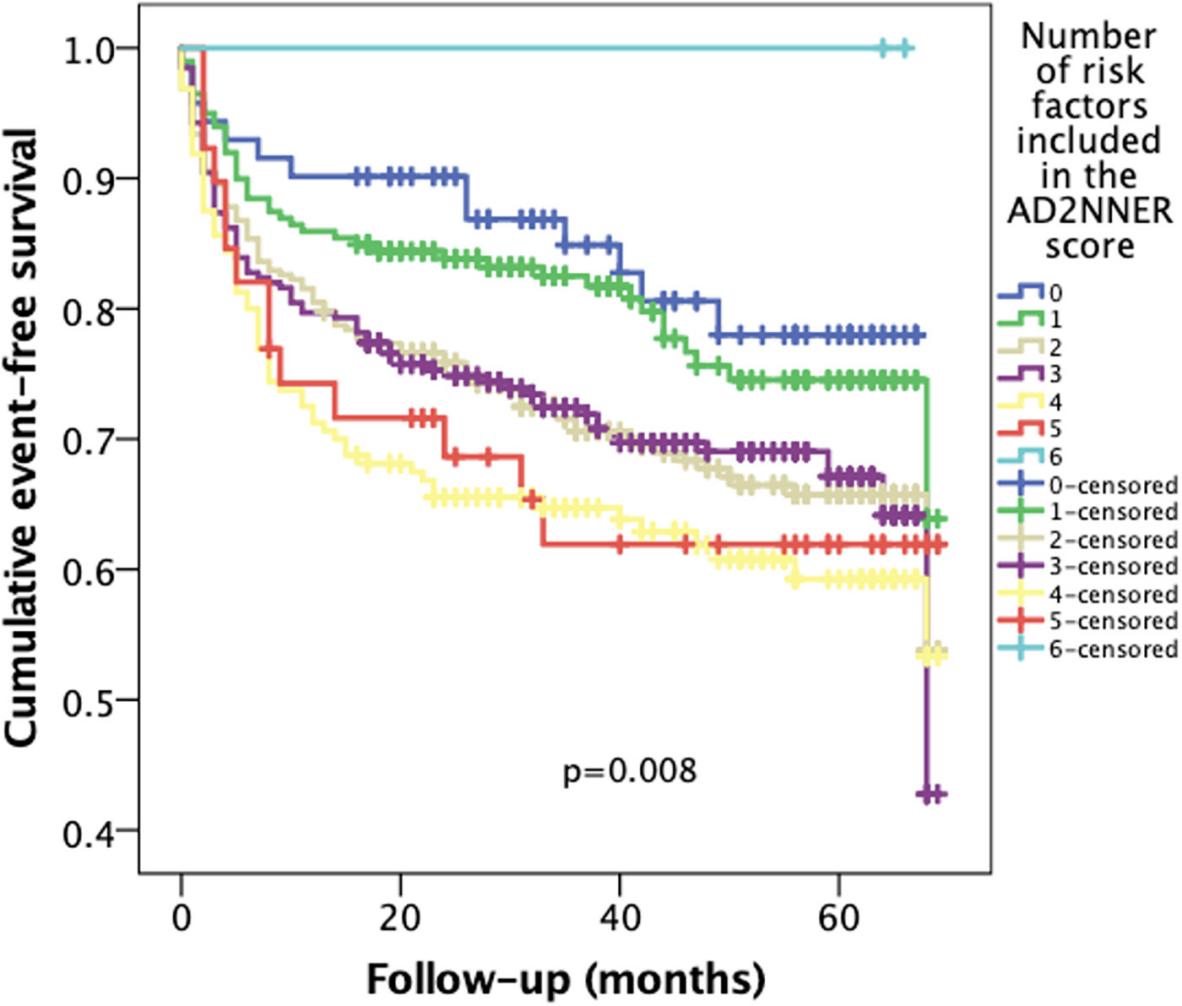
Kaplan–Meier curves for cumulative event-free survival according to the number of unweighted risk factors (0–6) included in the AD2NNER model.

In addition, a Kaplan–Meier analysis using the unweighted count of the six risk factors included in the AD2NNER score (age ≥75, NYHA III–IV, AF, T2DM, hyponatremia, and ischemic or congenital etiology) revealed a progressive decline in event-free survival across groups ranging from 0 to 6 risk factors. The differences between curves were statistically significant (*p* = 0.008), supporting the additive prognostic value of each individual variable (Figure 3). When visually compared to the AD2NNER score-based stratification between 0 and 9 (Figure 4), the risk count method demonstrated similar directionality, but with less refined differentiation, particularly in mid-risk strata. Among the three Kaplan–Meier models, the dichotomized AD2NNER score (0–3 vs. 4–9) provided the most distinct and clinically interpretable risk stratification.

**Figure 5:**
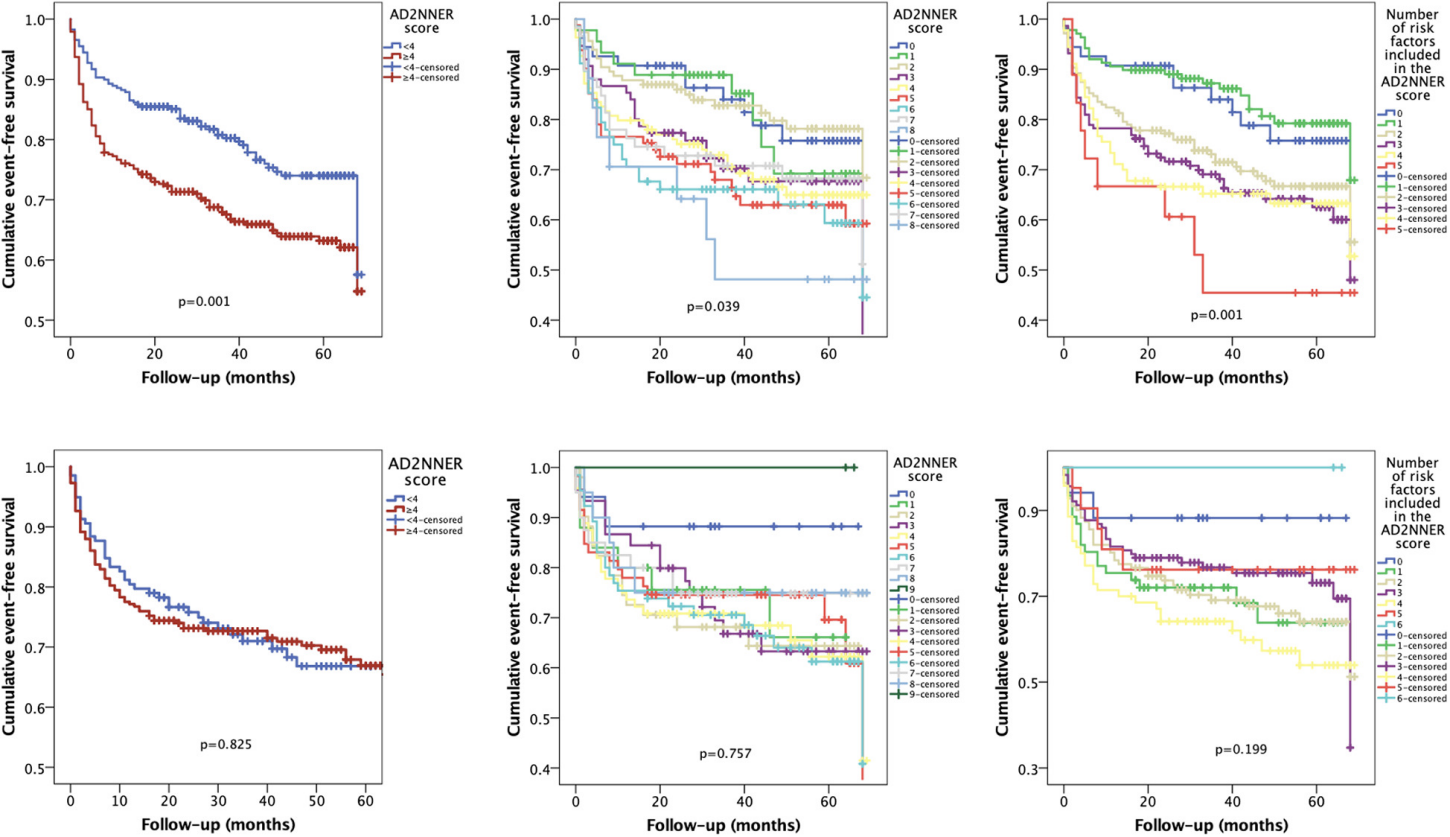
Kaplan–Meier curves for cumulative event-free survival stratified by the AD2NNER score in hFpEF and hFmrEF patients. Kaplan–Meier analyses were performed separately for patients with HFpEF (top row) and HFmrEF (bottom row), using the dichotomized AD2NNER score (left), the full score (0–9; center), and the unweighted risk factor count (0–6; right).

In subgroup analysis of patients with HFpEF (Figure 5, top row), the AD2NNER score showed good discriminatory performance for predicting rehospitalization. The dichotomized score (≥4 vs. <4) significantly stratified event-free survival (*p* = 0.001), as did both the full score (0–9; *p* = 0.039) and the unweighted number of risk factors (0–6; *p* = 0.001). In contrast, in patients with HFmrEF (Figure 5, bottom row), none of the Kaplan–Meier survival analyses reached statistical significance. The dichotomized AD2NNER score failed to separate groups (*p* = 0.825), as did the full score (*p* = 0.757) and the unweighted count (*p* = 0.199). These findings suggest that the AD2NNER score is more prognostically informative in patients with HFpEF than in those with HFmrEF.

### 3.4 Predictive value of the AD2NNER score and its components for all-cause mortality

During the follow-up period, mortality occurred in 89 (8.7%). In univariable logistic regression, NYHA class III–IV (OR = 1.86, 95% CI 1.16–2.97, *p* = 0.010), and ischemic or congenital etiology of HF (OR = 1.63, 95% CI 1.00–2.67, *p* = 0.049) were significantly associated with increased mortality. The remaining variables, including age ≥75 years, AF, T2DM, and hyponatremia, were not significantly associated with all-cause death.

In the multivariable logistic regression model, only NYHA class III–IV (OR = 0.58, 95% CI 0.36–0.95, *p* = 0.030) remained an independent predictor of mortality. The AD2NNER score, analyzed as a continuous variable (0–9), was significantly associated with mortality (OR = 1.18 per point, 95% CI 1.06–1.03, *p* = 0.002). When dichotomized at ≥4 points, it also remained predictive (OR = 2.21, 95% CI 1.35–3.62, *p* = 0.002).

Similarly, the unweighted risk factor count was a significant predictor of mortality (OR = 1.36 per additional risk factor, 95% CI 1.14–1.61, *p* = 0.001).

## 4 Discussion

### 4.1 Key findings

To the best of our knowledge, this study is the first to develop a multiparametric risk score for predicting rehospitalization in a large cohort of inpatients with HFpEF and HFmrEF by evaluating predisposing risk factors, clinical signs and symptoms, objective indicators of cardiac dysfunction, and both functional and structural abnormalities. The findings of our study can be summarized as follows: (i) Age, T2DM, NYHA class, ischemic or congenital etiology of HF, AF, and serum sodium levels were independently associated with the endpoint of HF rehospitalization; (ii) the prognostic model (the AD2NNER risk score) incorporating these variables effectively predicted the risk of rehospitalization in patients with HFpEF and HFmrEF; and (iii) patients with an AD2NNER score of 4 or higher demonstrated a significantly lower likelihood of event-free survival.

The progression of HF frequently follows an unpredictable trajectory. While some patients experience lifestyle-limiting symptoms due to elevated filling pressures during exercise but remain asymptomatic at rest, others develop intermittent fluid retention necessitating hospitalization. Patients with a history of hospitalization exhibit greater morbidity and mortality than those without prior hospitalizations and may respond differently to treatment. Furthermore, recurrent hospitalizations due to HF decompensation negatively impact long-term prognosis (12). The limited therapeutic options and suboptimal treatment responses in HFpEF and HmrEF patients may be attributed to their intricate and complex pathophysiology, the heterogeneity of the affected patient population, and the high prevalence of comorbidities at the time of diagnosis. Both the United States (13) and the European (4) HF guidelines underscore the necessity of addressing multiple comorbidities and tailoring interventions to the unique phenotypes of individual patients. Furthermore, identifying predictors and trends associated with HFpEF and HFmrEF readmission is a critical step toward achieving personalized management for this patient population. The literature largely overlooks the topic of HFpEF and HFmrEF in the context of acute presentations, with most existing data on HFpEF and HFmrEF outcomes originating from outpatient cohort studies, which may not accurately represent the prognosis of hospitalized patients with HF. Our study is among the few to focus specifically on hospital readmissions in inpatients. Additionally, previous studies identifying key predictors of HF readmission (14, 15) have focused predominantly on the general HF population rather than specifically on patients diagnosed with HFpEF and HFmrEF (16).

In our study, six factors were significantly associated with increased readmission rates: age, T2DM, NYHA class, ischemic or congenital etiology, AF, and serum natrium values. Advanced age is linked to declining health, reduced functional independence, polypharmacy, and a greater burden of comorbidities (17). T2DM increases the risk of HF by two- to fourfold (18), even in the absence of classical cardiovascular risk factors, coronary artery disease or valvular heart disease (19). While the percentage of diabetic individuals among HF patients is 36%, it appears to increase to 50% in decompensated HF patients, with these patients showing elevated in-hospital and one-year mortality, as well as a high rate of readmission. Nearly half of the patients who experienced the primary outcome in our study had T2DM. Furthermore, insulin resistance is strongly correlated with the NYHA functional class, a well-established predictor of HF rehospitalization (20), as confirmed in our cohort.

The high proportion of patients treated with beta-blockers (>80%) likely reflects adherence to guideline-recommended therapy for HFpEF and HFmrEF, particularly in those with comorbid conditions such as atrial fibrillation and hypertension. Regarding NT-proBNP levels, the absence of significant differences between rehospitalized and non-rehospitalized groups may reflect similar hemodynamic status at discharge, the influence of renal function, or the heterogeneity of HFpEF phenotypes. These results align with previous findings suggesting that while NT-proBNP remains an independent predictor in HFpEF, its prognostic utility is limited due to high variability and comorbidities in this population (21). While ischemic etiology is a primary cause of HFrEF, our results revealed a strong association between ischemic etiology and rehospitalization risk in patients with HFpEF and HFmrEF. However, coronary angiography was performed based on clinical judgment and indication, primarily in patients with symptoms or findings suggestive of ischemic heart disease. As a result, some patients with low clinical suspicion were not evaluated invasively, potentially leading to under-recognition of ischemic etiology. Furthermore, the number of patients who underwent revascularization after diagnostic angiography was not systematically recorded. These factors may have contributed to misclassification and could have attenuated the observed strength of association between ischemic etiology and rehospitalization risk.

Similarly, AF, a common comorbidity in HF patients, is associated with poorer outcomes (22) and significantly predicts HF rehospitalization in our study. Finally, hyponatraemia emerged as a significant predictor of the primary outcome, which is consistent with prior studies (23, 24).

We developed a risk prediction model for patients with HFpEF and HFmrEF that includes six clinically relevant variables into a scoring system ranging from 0 to 9, with a cutoff value of 4 providing the optimal prediction of the primary outcome—rehospitalization due to HF decompensation.

To evaluate the incremental value of the AD2NNER score beyond raw risk accumulation, we compared Kaplan–Meier curves generated using three different models: the full AD2NNER score (0–9), a simple unweighted risk factor count (0–6), and a dichotomized AD2NNER score (0–3 vs. 4–9). While both the full score and the unweighted count showed a stepwise decline in event-free survival, the full score failed to reach statistical significance, likely due to limited sample sizes in certain strata. The risk factor count demonstrated better separation (*p* = 0.008), confirming the additive contribution of individual clinical variables. However, the dichotomized AD2NNER score showed the clearest separation of survival curves (*p* = 0.005), offering both strong discriminatory power and ease of clinical application. This supports its potential utility as a pragmatic tool for risk stratification in patients with HFpEF or HFmrEF.

The significance and clinical utility of our risk score lies in its focus on morbidity among patients with an EF above 40%, a population often overlooked by existing models that mainly emphasize mortality across the full spectrum of EF or focus on patients with an EF above 50%.

Subgroup analyses further revealed that the prognostic value of the AD2NNER score was primarily driven by patients with HFpEF. In this group, the score—whether analyzed continuously, dichotomously, or via unweighted risk count—was consistently associated with event-free survival. Conversely, the score did not significantly stratify risk in the HFmrEF subgroup, where survival curves showed marked overlap. This differential performance may reflect underlying pathophysiologic differences between HFpEF and HFmrEF phenotypes, or potentially lower event rates and statistical power in the HFmrEF group. These findings highlight the importance of EF-specific validation when applying prognostic models in HF populations.

Finally, we also explored the association between the AD2NNER risk score and all-cause mortality. In our cohort, higher AD2NNER scores—both as continuous and dichotomized variables—were significantly associated with increased mortality, suggesting that the score may also reflect broader prognostic risk. Among the six included risk factors, NYHA functional class III/IV remained independently associated with all-cause mortality. The absence of time-to-death data precluded survival curve analysis or Cox regression, which limits interpretability. Nonetheless, these findings indicate that the AD2NNER score, although originally derived for rehospitalization risk, may also provide valuable mortality risk stratification in patients diagnosed with HFpEF and HFmrEF.

Furthermore, we recognize the need to clarify the added clinical value of our weighted score compared to a simpler unweighted approach. Although the unweighted risk factor model also demonstrated statistically significant associations with adverse outcomes, the AD2NNER score provides several distinct advantages that enhance its clinical utility. First, it incorporates variable weighting based on the magnitude of prognostic impact in multivariable analysis, offering a more nuanced assessment of risk than simple additive models. This is particularly relevant in patients with multiple coexisting high-impact predictors, such as advanced NYHA class and AF, where risk accumulation may not be linear. Second, the AD2NNER score allows for better differentiation in intermediate-risk groups, as illustrated in the Kaplan–Meier survival curves, where the dichotomized version (cutoff ≥4) provided clearer risk stratification than the unweighted count. Third, the weighting method facilitates clinical interpretation by highlighting which specific risk domains—congestive symptoms, rhythm abnormalities, metabolic status, or etiology—drive a patient's risk profile, supporting more tailored interventions. While the unweighted count showed slightly higher odds for mortality, the AD2NNER score was optimized for rehospitalization prediction, which was the primary endpoint. Importantly, the AD2NNER score also offers improved bedside applicability through a validated cutoff point that simplifies stratification without sacrificing interpretability. Thus, despite comparable statistical performance, the AD2NNER score presents conceptual, clinical, and pragmatic advantages that support its preferential use in HFpEF and HFmrEF populations.

### 4.2 Comparison with existing prognostic models

To the best of our knowledge, five risk scores have been developed for HFs with EFs above 40%: CHARM(5), MAGGIC(6), I-PRESERVE(7), 3A3B SCORE(8), and WATCH-DM(9).

Our study distinguishes itself by focusing on patients with both HFpEF and HFmrEF. The CHARM and MAGGIC risk scores included HF patients across the entire EF spectrum rather than the specific subgroups of HFpEF or HFmrEF. Conversely, scores such as 3A3B (26) and WATCH-DM included only patients with an EF above 50%, with WATCH-DM further limiting its scope to a selected population of patients with HFpEF and concurrent T2DM. Some variables, such as age (MAGGIC, 3A3B, I-PRESERVE), diabetes status (MAGICC, I-PRESERVE, WATCH-DM), NYHA class (MAGICC), sodium level (CHARM), HF etiology and cardiac rhythm, also stand out as valuable parameters for predicting hospitalization rates for patients with HFpEF and HFmrEF, overlap with previously mentioned scores.

Additionally, the MAGGIC score is based on data from studies conducted between 1980 and 2006, rendering it potentially less relevant to contemporary HF populations. Similarly, the CHARM risk score, developed over 15 years ago, may not fully capture the evolving characteristics of modern HF cohorts. This distinction is crucial, as the prevalence of valvular pathology, congenital heart disease in adults, and the use of implantable devices—key features of contemporary HF populations—were either excluded or underrepresented in these earlier scores. For example, MAGGIC excludes patients with valvular pathology greater than moderate severity, a group increasingly represented in current clinical practice. In contrast, we included patients with a wide range of HF etiologies.

The AD2NNER risk score also stands out by incorporating a combination of clinical variables and laboratory tests that are, however, accessible and practical assessments, unlike complex models such as I-PRESERVE, which include variables not routinely assessed in clinical practice (i.e., quality-of-life scores). On the other hand, the CHARM and MAGGIC risk scores omit routinely assessed and valuable parameters such as the underlying cardiac rhythm or HF etiology.

Recent developments in machine learning (ML) have introduced advanced phenotyping and risk stratification methods in HFpEF and HFmrEF populations. These approaches can handle high-dimensional data and identify complex, non-linear relationships, as shown in a recent review (25). However, ML models often lack transparency and require significant computational infrastructure, limiting their real-world applicability. In contrast, the AD2NNER score offers a clinically intuitive and easily implementable model using routinely available variables, while still providing meaningful prognostic discrimination.

Summarizing, the AD2NNER risk score represents a promising advancement in stratifying rehospitalization risk among patients with HFpEF and HFmrEF. By integrating readily accessible clinical and laboratory parameters, this model fills a critical gap left by earlier risk scores and offers a practical tool for personalized patient management. Compared to an unweighted count of risk factors, the AD2NNER score allows for more nuanced risk differentiation, particularly among intermediate-risk patients. Its use of weighted variables enhances discrimination, while the dichotomized version (cutoff ≥4) supports rapid bedside decision-making. This balance between simplicity and predictive strength increases its applicability in clinical workflows. Future multicenter studies and prospective validations are warranted to refine the score further and confirm its clinical utility. Ultimately, incorporating such risk stratification tools into routine practice may guide targeted interventions, enhance patient outcomes, and alleviate the overall burden of HF rehospitalizations.

### 4.3 Limitations

We acknowledge several limitations of our study that should be considered when the findings are interpreted. First, owing to its retrospective design, it can include potential selection bias and incomplete data from electronic health records; our study did not account for all potential confounding variables. Second, advanced echocardiographic data with demonstrated prognostic value in patients with HF were not included. Third, the heterogeneity of HFpEF and HFmrEF has not been fully addressed, and detailed phenotyping for diverse clinical subtypes and etiologies is necessary. Finally, as this was a single-center study, our results should be validated in larger, prospective multicenter studies.

## 5 Conclusion

The AD2NNER risk score is a simple, six-variable model that accurately predicts rehospitalization in a large cohort of inpatients with HFpEF and HFmrEF, with a cutoff value of 4. This tool offers clinicians a practical and efficient method to identify high-risk patients, enabling targeted interventions to potentially reduce rehospitalization rates and improve outcomes. In addition, higher AD2NNER scores were associated with all-cause mortality, suggesting broader prognostic relevance.

## Data Availability

The raw data supporting the conclusions of this article will be made available by the authors, without undue reservation.
